# Maternal and perinatal outcome of preeclampsia without severe feature among pregnant women managed at a tertiary referral hospital in urban Ethiopia

**DOI:** 10.1371/journal.pone.0230638

**Published:** 2020-04-09

**Authors:** Lemi Belay Tolu, Endale Yigezu, Tadesse Urgie, Garumma Tolu Feyissa

**Affiliations:** 1 Saint Paul’s Hospital Millennium Medical College, Addis Ababa, Ethiopia; 2 Department of Health, Behavior and Society, Jimma University, Jimma, Ethiopia; University of Mississippi Medical Center, UNITED STATES

## Abstract

**Background:**

Preeclampsia refers to the new onset of hypertension and proteinuria after 20 weeks of gestation in a previously normotensive woman. Pregnant women with preeclampsia are at an increased risk of adverse maternal, fetal and neonatal complications. The objective of the study is, therefore, to determine the maternal and perinatal outcome of preeclampsia without severity feature among women managed at a tertiary referral hospital in urban Ethiopia.

**Methods:**

A hospital-based prospective observational study was conducted to evaluate the maternal and perinatal outcome of pregnant women who were on expectant management with the diagnosis of preeclampsia without severe feature at a referral hospital in urban Ethiopia from August 2018 to January 2019.

**Results:**

There were a total of 5400 deliveries during the study period, among which 164 (3%) women were diagnosed with preeclampsia without severe features. Fifty-one (31.1%) patients with preeclampsia without severe features presented at a gestational age between 28 to 33 weeks plus six days, while 113 (68.9%) presented at a gestational age between 34 weeks to 36 weeks. Fifty-two (31.7%) women had maternal complication of which, 32 (19.5%) progressed to preeclampsia with severe feature Those patients with early onset of preeclampsia without severe feature were 5.22 and 25.9 times more likely to develop maternal and perinatal complication respectively compared to late-onset after 34 weeks with P-value of <0.0001, (95% CI 2.01–13.6) and <0.0001(95% CI 5.75–115.6) respectively.

**Conclusion:**

In a setting where home-based self-care is poor expectant outpatient management of preeclampsia without severe features with a once per week visit is not adequate. It’s associated with an increased risk of maternal and perinatal morbidity and mortality. Our findings call for special consideration and close surveillance of those women with early-onset diseases.

## Introduction

Preeclampsia is defined as a systemic syndrome characterized by the new onset of raised blood pressure >140/90 mm Hg and proteinuria after 20 weeks of gestation in a previously normotensive woman [1, 2]. Globally preeclampsia complicates 2–8% of pregnancies and contributes to 10–15% maternal death [3]. It’s called preeclampsia without severity feature in the absence any of the following features: cerebral symptoms (like visual disturbance, headache), right upper quadrant or epigastric pain, serum transaminase concentration ≥ twice normal, systolic blood pressure ≥160 mm Hg, and or diastolic blood pressure ≥110 mm Hg on two occasions at least four hours apart, severe thrombocytopenia (<100,000 platelets/micro), Oliguria <500 mL in 24 hours and pulmonary edema [2, 4, 5].

Multiple observational studies reported a prevalence of preeclampsia in Ethiopia ranging from 4 to 12% and contribute to 15% maternal deaths [6–9]. In five year’s retrospective review of the perinatal outcome at three teaching hospitals in Ethiopia, preeclampsia contributed to perinatal mortality of 290/1000 total births [10].

The only curative treatment of preeclampsia is birth. However, in the case of preterm pregnancies, expectant management is advocated to increase the chance of fetal maturity, if the risk for the mother remains acceptable [11]. The Hypertension and Preeclampsia Intervention Trial At near Term (HYPITAT) which is a multicenter RCT comparing expectant management versus induction of labour in a woman with mild gestational hypertension or mild preeclampsia at 36 to 37 weeks of gestation has shown that routine induction was associated with a significant reduction in composite adverse maternal outcome without affecting the neonatal outcome [12]. An observational study has also shown that the onset of mild gestational hypertension or mild preeclampsia at or near term is associated with minimal to low maternal and fetal complications [13].

World Health Organization (WHO) recommends expectant management of preeclampsia without severity feature until 37 weeks [14]. In Ethiopia in general and Saint Paul’s Hospital Millennium Medical College (SPHMMC), preeclampsia without severity features is managed expectantly until 37 weeks. At SPHMMC preeclampsia without severity feature is managed as an outpatient with once per week visit. Patients will visit their physician once per week and evaluated for any severe features by history, blood pressure(BP) measurement, laboratory evaluation, and obstetric ultrasound.

The justification for expectant management was the risk of increased assisted vaginal delivery, cesarean section and prematurity, and its complication, thus generating additional morbidity and cost [15]. On the other hand, there is the possibility of progression of the preeclampsia without severe feature to preeclampsia with severity feature leading to eclampsia, severe hypertension, abruption, pulmonary edema, HEELP (Hemolysis, Elevated liver Enzymes and Low Platelet) syndrome and adverse neonatal outcome [5, 16–18].

The optimal management of preeclampsia without severe features remains controversial especially in developing countries like Ethiopia where home-based self-care like blood pressure monitoring is barely possible. Limited studies suggest that patients offered outpatient monitoring should be able to comply with frequent maternal and fetal evaluations, some form of blood pressure monitoring at home and should have ready access to medical care [4, 19, 20]. There is no data on maternal and perinatal outcomes of preeclampsia without severity feature in Ethiopia with current outpatient management by once per week visit. The aim of the study was; therefore, to determine the maternal and perinatal outcome of expectantly managed pregnant women with a diagnosis of preeclampsia without severity feature between gestational age of 28 and 36 completed weeks at the tertiary teaching hospital in Addis Ababa, Ethiopia.

## Materials and methods

The study was conducted at Saint Paul’s Hospital Millennium Medical College (SPHMMC), Addis Ababa, Ethiopia from August 1, 2018, to January 31, 2019. SPHMMC is a tertiary teaching referral hospital giving the highest maternity service in Ethiopia. The hospital handles complicated obstetric cases and often referring normal or less complicated pregnant mothers to other hospitals or health centers for birth. According to the statistics office of the hospital, 9000 births were attended in 2018,35% of births were by cesarean section. This was a prospective non-comparative observational study conducted to determine the maternal and perinatal outcome of expectantly managed pregnant women with a diagnosis of preeclampsia without severity feature among women receiving Antenatal care (ANC) and birthing at Saint Paul’s Hospital Millennium Medical College. Preeclampsia without severity feature was the exposure variable. Outcome variables considered in this study were admission to the neonatal intensive care unit (NICU) and adverse maternal and perinatal outcomes. Confounding variables were socio-demographic characteristics (maternal age, educational status, occupation, residency) and obstetrics variables (Gravidity, mode of delivery, gestational age, ANC visit).

### Operational definitions

Preeclampsia without severe feature: raised BP ≥ 140/90 mmHg plus 24-hour urine protein greater than or equal to 300mg/24 hour or urine dipstick >+1 after 20 weeks of gestation in previously normotensive women [2].

Adverse perinatal outcome: pregnancy outcome including stillbirth, growth restriction (IUGR), respiratory distress syndrome (RDS), low birth weight, low 5^th^ minute APGAR score, neonatal death.

Stillbirth: fetus born with no sign of life after 28 weeks of gestational age.

Adverse maternal outcome: pregnancy complicated by severe preeclampsia [2] with one of the following features: cerebral symptoms (like visual disturbance, headache), right upper quadrant or epigastric pain, serum transaminase concentration ≥ twice normal, systolic blood pressure ≥160 mm Hg, and or diastolic blood pressure ≥110 mm Hg, severe thrombocytopenia (<100,000 platelets/micro), acute kidney injury and pulmonary edema. Eclampsia, Disseminated Intravascular Coagulation(DIC), abruption, HEELP (Hemolysis, Elevated liver Enzymes, and Low Platelet) syndrome, and maternal death was also considered as an adverse maternal outcome.

Expectant management: Women who have at least one visit at SPHMMC with an established diagnosis of preeclampsia without severe features between 28–36 weeks of gestational age.

Early-onset preeclampsia without severe feature: preeclampsia without severe feature diagnosed at a gestational age between 28–34 weeks.

Late-onset preeclampsia without severe feature: preeclampsia without severe feature diagnosed at a gestational age between 34–36 weeks.

The study Included all consented pregnant women, irrespective of the number of gestations, with the diagnosis of preeclampsia without severe feature at a gestational age of 28 weeks to 36 weeks who had at least one visit and managed at SPHMMC from August 1/2018 to January 31/2019. Pregnant women with gestational age less than 28 weeks and greater than 36 weeks, those with other types of hypertensive disorder of pregnancy like preeclampsia with severity feature, gestational hypertension, eclampsia, superimposed preeclampsia, chronic hypertension, and patients with known fetal lethal congenital anomalies were excluded from the study. Pregnant mothers with comorbid chronic medical disorders like diabetes, severe anemia, renal disease, cardiac disease, antiphospholipid antibody syndrome and those with known TORCH infections were also excluded.

Data were collected by trained midwives at regular ANC units, emergency unit and labour ward and nurse at NICU (Neonatal Intensive Care Unit) using a pre-tested structured questionnaire. At initial visit data collectors at regular ANC enrolled those mothers who consented and diagnosed to have preeclampsia without severe features based on criteria and then documented their socio-demographic and obstetrics characters. Card and phone number of mothers were also registered for later tracing of maternal and perinatal outcomes. Women were followed till birth and data regarding maternal and perinatal outcomes was collected using a well-structured questionnaire by observation and from the maternal and neonatal cards at the birth unit and NICU including the first week after delivery. For those discharged home maternal and neonatal condition was checked on the seventh day during follow up and those who didn’t appear on follow up day were reminded by cell phone call.

After data collection, data cleaning was performed to check for outliers, missed values, and any inconsistencies. Data were entered and analyzed using SPSS version 23. Descriptive statistics (frequency and proportions) were used to characterize the variables and determine the proportion of maternal and perinatal outcomes of preeclampsia without severe features. Bivariate and multivariate logistic regression was used to see the association of variables and to identify independent factors affecting maternal and perinatal outcomes and control confounders. Odds Ratio (OR),95% confidence interval and a P-value set at 0.05 were used to determine the statistical significance of the association.

### Ethical consideration

Ethical approval was obtained from Saint Paul’s Hospital Millennium Medical College ethical review committee. Written informed consent to conduct a study and publish the outcome was obtained from each patient and confidentiality was maintained during data collection, analysis, and interpretation. All the datasets used and/or analyzed during the current study are included in the manuscript and available from the corresponding author on reasonable request.

## Results of the study

### Socio-demographic and obstetric characteristics of participants

There was a total of 5400 births at SPHMMC during the study period of which 164 (3.0%) of them were women managed with the diagnosis of preeclampsia without severe features. In this cohort of women diagnosis of preeclampsia without severe feature was made by BP and urine dipstick for protein >+1 in 102 (62.2%) women. The remaining 62 (37.8%) of women were diagnosed to have preeclampsia with raised BP and twenty-four-hour urine protein excretion greater than 300 mg. Gestational age at the time of diagnosis was between 28–36 weeks and there was no loss to follow up.

Most of the women 99 (60.4%) were aged 20–34 years and lived in Addis Ababa 117 (71.3%). Most women were multigravida 84 (51.2%) and the gestation at first presentation was predominantly between 34+0 weeks and 36+6 weeks. Table 1 describes the Socio-demographic and obstetric characteristics of the cohort. (Table 1).

**Table 1:** Socio demographic and obstetric characteristics of mothers on expectant management with a diagnosis of preeclampsia without severe feature at SPHMMC,2018 (N = 164).

Variable.	Category.	Number of patients (%)
Religion.	Orthodox	75(45.7%)
	Muslim	67(40.9)
	Protestant	20(12.2%)
	Catholic	2(1.2%)
Age(years)	<20	41(25%)
	20–34	99(60.4%)
	35–49	24(14.6%)
Marital status	Married	146(89%)
	Single	15(9.1%)
	Divorced	3(1.9%)
Educational status	No formal education	49(29.9%)
	Primary	89(54.3%)
	Secondary	23(14%)
	College and above	3(1.8%)
Occupation	Government employee	23(14%)
	Merchant	43(26.2%)
	Housewife	81(49.3%)
	Daily laborer	17(10.5%)
Residency	From Addis Ababa.	117(71.3%)
	Rural.	47(28.7%)
Gravidity	Primigravida.	80(48.8%)
	Multigravida.	84(51.2%)

**Fig 1:**
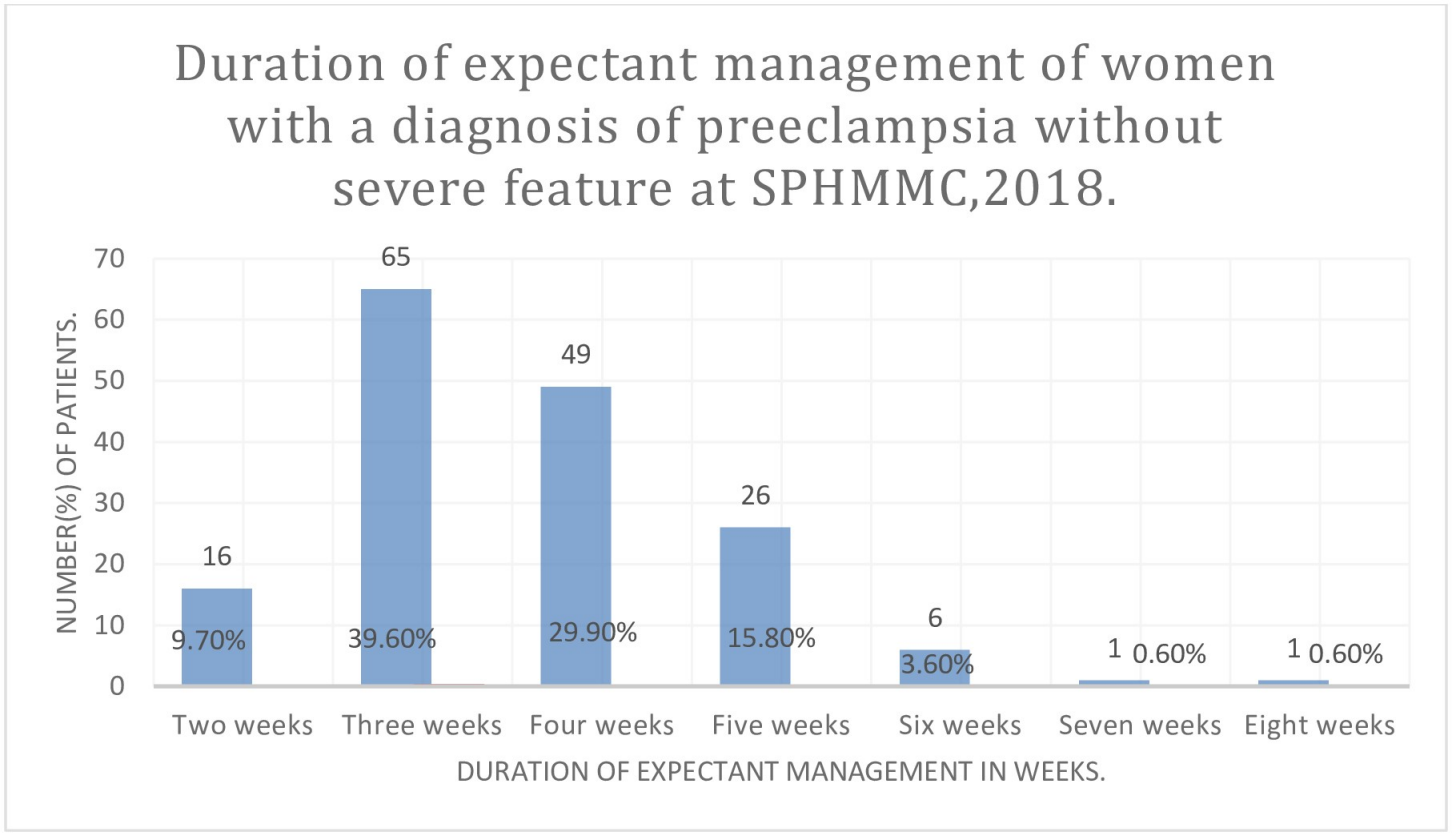
Duration of expectant management of women with a diagnosis of preeclampsia without severe feature at SPHMMC, 2018.

The mean duration of expectant management was 4.6 weeks. The follow-up duration ranges from two weeks to eight weeks. Figure one describes the duration of expectant management in weeks with a respective number of women (Fig 1).

### Maternal and perinatal outcomes of the study population

One third (52, 31.7%) of the women had maternal complications of which 32 (19.5%) progressed to preeclampsia with a severe feature. There were two (1.22%) maternal deaths. Thirty-two (19.5%) of women delivered before 37 weeks. Twenty-two (12.5%) of neonates had a birth weight of less than 2.5 kg. Sixty (36.6%) neonates were admitted to NICU. There were three (1.7%) stillbirths and four (2.27%) early neonatal deaths. The perinatal mortality rate was 4.26% (42.6/1000). Five (71%) of perinatal deaths occurred in preterm births. Table 2 describes the maternal, fetal and neonatal adverse outcomes.

**Table 2:** Maternal, fetal and neonatal complications of expectantly managed preeclampsia without severe feature at SPHMMC, 2018.

Outcome measures	Frequency	Percent (%)
1. Maternal complications		
Preeclampsia with severity	32	19.5
Placental abruption	9	5.4
Partial/complete HEELP	4	2.43
Eclampsia	2	1.22
DIC	2	1.22
Maternal death	2	1.22
2. Fetal complications		
Stillbirth.	3	1.7
Intrauterine growth restriction(IUGR).	22	12.5
Preterm birth	32	18.18
3. Neonatal complications.		
NICU admission	60	36.6
Low APGAR score.	4	2.27
Respiratory distress syndrome(RDS).	7	3.97
Early neonatal death(END).	4	2.27

### Factors associated with adverse maternal and perinatal outcomes

Occupation, religion, marital status, educational status and place of residency are not associated with poor maternal and perinatal outcomes. There is no significant difference in the rate of adverse maternal and perinatal outcomes between primigravid and multigravida women. Women older than 35 years were 2.54 times more likely to develop adverse maternal outcomes compared to those in the middle age group (20–35) with a P-value of 0.030, (95%CI 1.021–6.32). Those patients with early onset of preeclampsia without severe feature were 5.22 and 25.9 times more likely to develop maternal and perinatal complication respectively compared to late-onset preeclampsia after 34 weeks with P-value of <0.0001, (95% CI 2.01–13.6) and <0.0001, (95% CI 5.75–115.6) respectively (Table 3).

**Table 3:** Factors associated with adverse maternal and perinatal outcomes during expectant management of preeclampsia without severe feature at SPHMMC, 2018.

Character.	Adverse maternal outcome.		COR(95%Cl)	AOR(95%Cl)	P-value
	Yes	No			
Age					
<20years	16	25	1,63(0.75–3.48)	1.26(0,56–2.81)	0.2147
20–34 years	28	71	1	1	
>35 years	12	12	2.54(1.02–6.32)	1.56(1.01–2.77)	0.030
Gravidity					
Primigravida	31	49	1.493(0.78–2.846)	1.704(0.84–3.45)	0.2260
Multi gravida	25	59	1	1	
Gestational age					
28–34 weeks	19	14	4.99(2.24–11.12)	5.22(2.01–13.6)	0.0001
34–36 weeks	28	103	1	1	
Character.	Adverse perinatal outcome				
	Yes	No			
Age					
<20years	24	21	2.22(0.92–4.54)	1.41(0.66–3.03)	0.0285
20–34 years	35	68	1	1	
>35 years	13	15	1.68(0.72–3.93)	1.12(0.68–1.74)	0.2281
Gravidity					
Primigravida	40	52	1.25(0.65–2.3)	1.24(0.63–2.35)	0.4684
Multigravida	32	52	1	1	
Gestational age					
28–33 weeks + 6 days.	33	8	10.15(4.31–23.87)	25.9(5.75–115.6)	<0.0001
34–36 weeks	39	96	1	1	

## Discussion

The current study is peculiar in that deals it with preeclampsia without severity feature alone which is unlike previous studies [10, 21–24]. The incidence of preeclampsia without severe features in this study was about 3.0% and 60.4% of women in the current study were in the age group 20–34. This finding is similar to other studies [7, 9, 22].

In this study gestational age at the time of diagnosis was between 34–36 weeks in 68.9% of the participants and 31.1% of the pregnant women presented at a gestational age between 28–33 weeks and 6 days. This finding is comparable to a study conducted in India that showed the incidence of late-onset preeclampsia (beyond 34 weeks) to be 72.4% and early-onset preeclampsia 27.6% with higher maternal and perinatal complications in early-onset preeclampsia [22].

The average duration of expectant management was 4.6 weeks and 34.1% of women developed one or more of the maternal, fetal or neonatal complications. In our study maternal complication was seen in 31.7% These complications were: progression to severe preeclampsia (18.2%), placental abruption (5%), HELLP syndrome (2.4%) and DIC (1.22%). These complications are high compared to a retrospective analysis of the incidence of severe disease in mild preeclampsia in China which showed 6% of preeclampsia women developed one of the following severe features: placental abruption (2.8%), eclampsia (0.9%%) and HELLP syndrome (0.6%) [25] There were two (1.22%) cases of eclampsia and maternal death in the current study which was high for women on follow up compared to similar study [25], which might imply outpatient follow up with once per week visit is inadequate for early identification and management of progression to severe preeclampsia.

The perinatal mortality in our study was 42.6 per 1000, while the overall perinatal complication rate was about 40.9%. Preterm birth was the commonest perinatal complication observed in this study accounting for 18.2%., Intrauterine growth restriction occurred in 12% of the cases, stillbirth occurred in about 1.7% and 2.27% of newborn ended up in early neonatal death. This finding is comparable to other study findings [22, 26], but since these studies are reporting on cumulative preeclampsia it’s difficult to make an exact comparison.

Consistent with other studies [27–29], the current study showed early onset of preeclampsia without severe feature was associated with increased risk of developing adverse maternal-fetal/neonatal outcomes compared to late-onset after 34 weeks. The increased perinatal complication seen might be explained by the progression of preeclampsia to severe diseases in those women who developed preeclampsia before 34 weeks and concomitant high preterm birth [30, 31]. Women older than 35 years have 2.54 times increased chance of having an adverse maternal outcome in this study compared to those in the middle age group (20–35).

The current study has limitations. Having gestational age-matched non-exposed (without preeclampsia) group would have been important to controlling confounders like the quality of care and preterm birth associated morbidity and mortality.

## Conclusion

In a setting where home-based self-care is poor expectant outpatient management of preeclampsia without severe features with a once per week visit is not adequate. It’s associated with an increased risk of maternal and perinatal morbidity and mortality. Our findings call for special consideration and close surveillance of those women with early-onset diseases.

## Supporting information

S1 Checklist Strobe checklist for observational studies. (DOCX)

## Abbreviations

ANC: Ante-Natal Care
APGAR: Appearance, Pulse, Grimace, Activity, Respiration
DIC: Disseminated Intravascular Coagulation
END: Early Neonatal Death
HELLP: Hemolysis Elevated Liver Enzyme Platelet
HYPITAT: Hypertension and Preeclampsia Intervention Trial at near
IRB: Institutional Review Board
IUGR: Intrauterine Growth Restriction
LBW: Low Birth Weight
NICU: Neonatal Intensive Care Unit
RCT: Randomized Control Trial
RDS: Respiratory Distress Syndrome
SPHMMC: Saint Paulo’s Hospital Millennium Medical College
SPSS: Statistical Package of Social science
WHO: World Health Organization
